# Symptoms Prompting Interest in Celiac Disease and the Gluten-Free Diet: Analysis of Internet Search Term Data

**DOI:** 10.2196/13082

**Published:** 2019-04-08

**Authors:** Benjamin Lebwohl, Elad Yom-Tov

**Affiliations:** 1 Celiac Disease Center Columbia University New York, NY United States; 2 Microsoft Research Herzeliya Israel; 3 Technion Haifa Israel

**Keywords:** celiac disease, gluten, epidemiology

## Abstract

**Background:**

Celiac disease, a common immune-based disease triggered by gluten, has diverse clinical manifestations, and the relative distribution of symptoms leading to diagnosis has not been well characterized in the population.

**Objective:**

This study aimed to use search engine data to identify a set of symptoms and conditions that would identify individuals at elevated likelihood of a subsequent celiac disease diagnosis. We also measured the relative prominence of these search terms before versus after a search related to celiac disease.

**Methods:**

We extracted English-language queries submitted to the Bing search engine in the United States and identified those who submitted a new celiac-related query during a 1-month period, without any celiac-related queries in the preceding 9 months. We compared the ratio between the number of times that each symptom or condition was asked in the 14 days preceding the first celiac-related query of each person and the number of searches for that same symptom or condition in the 14 days after the celiac-related query.

**Results:**

We identified 90,142 users who made a celiac-related query, of whom 6528 (7%) exhibited sustained interest, defined as making a query on more than 1 day. Though a variety of symptoms and associated conditions were also queried before a celiac-related query, the maximum area under the receiver operating characteristic curve was 0.53. The symptom most likely to be queried more before than after a celiac-related query was diarrhea (query ratio [QR] 1.28). Extraintestinal symptoms queried before a celiac disease query included headache (QR 1.26), anxiety (QR 1.10), depression (QR 1.03), and attention-deficit hyperactivity disorder (QR 1.64).

**Conclusions:**

We found an increase in antecedent searches for symptoms known to be associated with celiac disease, a rise in searches for depression and anxiety, and an increase in symptoms that are associated with celiac disease but may not be reported to health care providers. The protean clinical manifestations of celiac disease are reflected in the diffuse nature of antecedent internet queries of those interested in celiac disease, underscoring the challenge of effective case-finding strategies.

## Introduction

Celiac disease is a multisystem immune-based enteropathy characterized by autoantibodies to tissue transglutaminase and villous atrophy that is triggered by the ingestion of gluten in genetically predisposed individuals [1]. Present in nearly 1% of the US population, the seroprevalence of celiac disease has risen markedly in recent decades [2]. However, this rise in celiac disease prevalence has been eclipsed by a more dramatic rise in the avoidance of gluten among individuals who do not have celiac disease or have not been tested for celiac disease [3]. The reasons for gluten avoidance are manifold and include gastrointestinal symptoms [4], the avoidance of cardiometabolic complications [5], cognitive health [6], and treatment of autoimmune disease [7].

Although classical celiac disease consists of a malabsorption phenotype characterized by diarrhea and weight loss, the majority of patients with celiac disease are diagnosed with a *nonclassical* presentation that includes a heterogeneous set of symptoms and signs including osteoporosis, anemia, abnormal liver enzymes, neuropathy, infertility, and others [8]. Given these diverse clinical manifestations and the lack of signs and symptoms that are sensitive and specific for celiac disease, diagnosis can be elusive, and there is frequently a long delay between the onset of symptoms and the diagnosis of celiac disease. In 1 survey of adults in the United States, patients with celiac disease had symptoms for a mean of 11 years before diagnosis [9]. Patients with extraintestinal symptoms tend to have a longer diagnostic delay compared with those with intestinal symptoms [10].

Aside from clinical examination, researchers have used electronic medical records to screen for celiac disease, showing that analysis of free text included therein could aid in the early discovery of celiac disease [11]. Recently, another novel method for the ascertainment of symptoms preceding diagnoses has been proposed through the analysis of search engine queries [12]. For instance, search engine query analysis has been used to identify symptoms of pancreatic adenocarcinoma months before the disease diagnosis [13]. In this study, we examined search query data with the aim of identifying symptoms and conditions that are associated with a subsequent query for celiac disease and celiac disease–specific searches. We hypothesized that searches related to the modes of presentation of celiac disease would precede searches for celiac disease and/or the gluten-free diet. We aimed to measure the relative prominence of these search terms before versus after a search related to celiac disease. We also aimed to identify a set of symptoms and conditions that would identify individuals at elevated likelihood of a subsequent celiac disease diagnosis.

## Methods

We extracted all English-language queries submitted to the Bing search engine between January 1, 2017, and October 31, 2017, by people in the United States. For each query, we extracted the time and date of the query, its text, an anonymous user identifier, and the zip code of the asker. Bing data are estimated to be a representative sample of US internet users [14].

Celiac-related queries (CRQs) were those queries that contained the words “celiac” or “gluten.” The queries were filtered to include only those queries by users who were active since at least September 1, 2017, and used CRQs during the month of October 2017, but not in the previous 9 months.

In addition, we identified queries that could indicate celiac disease by finding those queries that contained 1 or more of the following terms: marsh score, duodenal biopsy, intestinal biopsy, beyond celiac, celiac disease foundation, tissue transglutaminase (also as an acronym: TTG), gliadin antibody, celiac clinical trials, celiac trials, or gluten trials.

Some people identified themselves in their queries as having celiac disease, through queries such as “I have celiac disease, can I eat rice?”. To identify these self-identified users [15], we found all mentions of “I have celiac” or “I was diagnosed with celiac” and manually inspected each to exclude irrelevant queries (eg, “do I have celiac?”). Obviously, not all people who have celiac identify themselves in their queries, but this subset of the population is likely composed of celiac patients, and we calculated the prevalence of CRQs in this subset.

We defined people with a passing interest in celiac disease as those who made CRQs during only 1 day. This contrasts with people with a sustained interest who made CRQs over more than 1 day.

Symptoms mentioned in queries were identified by matching the text of queries to a list of 195 symptoms and their synonyms, as developed by Yom-Tov and Gabrilovich [16]. Similarly, medical conditions were found by extracting all 5521 diseases and their synonyms that appear in Wikipedia [17].

Recipe searches provide a representative sample of the dietary consumption of individuals [18]. Therefore, to evaluate the changes in diet made by people with CRQs, we followed the methodology used previously [18] to identify queries for recipes and map them to ingredients therein.

We attempted to identify users with sustained interest from all users using their queries. To do this, we represented each user by the number of times they queried for each symptom and each medical condition before the first CRQ. A predictive model using either linear regression or random forest with 50 trees was constructed and tested using 10-fold cross-validation [19].

This study was approved by the Behavioral Sciences Research Ethics Committee of the Technion, approval number 2018-032.

## Results

Of 90,142 users with at least 1 CRQ, 83,614 users (93%) were found with passing interest and 6528 (7%) exhibited sustained interest. Of the 6528 people who had a sustained interest in celiac disease, 104 (1.6%) entered at least one celiac indicator (see Table 1) compared with 336 (0.7%) who had a passing interest and 0.001% in the general population of Bing users.

**Table 1:** Symptoms and conditions that appear with the highest probability in the 14 days before the first celiac-related query compared with the 14 days after it in the entire population and in the sustained user population.

Category	All celiac queries		Sustained celiac queries	
	Item	Ratio	Item	Ratio
Symptoms	Steatorrhea	1.88	Diarrhea	1.34
	Dyspepsia	1.76	Cough	1.28
	Exophthalmos	1.63	Headache	1.27
	Vomiting	1.62	Bloating	1.26
	Stomach ache	1.57	Anxiety	1.10
	Diarrhea	1.50	Bleeding	1.08
	Xerostomia	1.49	Weight loss	1.03
	Flatulence	1.48	Depression	1.03
	Abdominal pain	1.46	Pain	1.01
	Bloating	1.45	Itch	1.00
Condition	Lactose intolerance	3.05	Autoimmunity	3.21
	Inflammatory bowel disease	2.58	Attention-deficit hyperactivity disorder	1.64
	Malabsorption	2.32	Hypothyroidism	1.38
	Peptic ulcer	2.22	Gastroesophageal reflux disease	1.33
	Irritable bowel syndrome	2.19	Asthma	1.30
	Food intolerance	2.10	Influenza	1.24
	Crohn disease	2.02	Migraine	1.22
	Digestive disease	1.96	Colitis	1.21
	Polycystic ovary syndrome	1.95	Systemic lupus erythematosus	1.13
	Peritonitis	1.92	Alzheimer disease	1.13

Only 31 users identified themselves as having celiac disease based on a self-identified query (eg, “I have celiac”). Among people with a sustained interest in celiac disease, 0.12% (8/6528) were those who identified themselves as having celiac disease compared with 0.03% (31/90142) in the general population (a ratio of 3.5). Thus, similar to the findings of Ofran et al [20], a sustained interest in celiac disease can be considered a proxy for having the condition or for a caregiver of a patient.

We attempted to identify users with either a passing or sustained interest in celiac disease, comparing them with all other users based on antecedent queries. In both cases, the area under the receiver operating characteristic curve was 0.53 or less, indicating that we could not distinguish the 2 classes based on symptoms and conditions searched.

To investigate the symptoms most associated with initiation of search for CRQs, we compared the ratio between the number of times that each symptom or condition were asked in the 14 days preceding the first CRQ of each person and the number of times they were mentioned from 14 days before the first CRQ until 14 days after it. Table 1 shows the symptoms and conditions with the highest before-to-after ratio.

**Figure 1:**
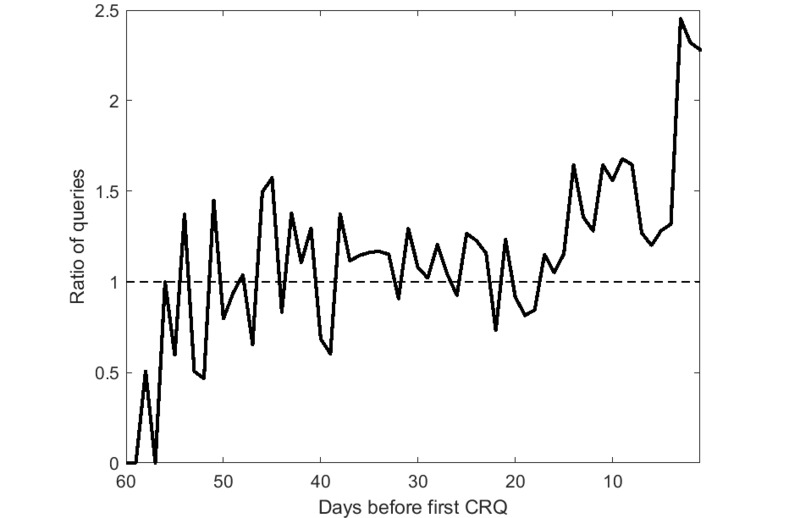
Fraction of queries for diarrhea over time, compared with the fraction of all queries. Day zero is the first celiac-related query. A ratio greater than 1 indicates that the queries for diarrhea are more common than could be expected. CRQ: celiac-related query.

Figure 1 shows the fraction of queries for “diarrhea,” the top-ranked symptom among sustained users, over time compared with the fraction of all queries. As the figure shows, interest in this symptom begins to rise only approximately 2 weeks before the first CRQ and rises dramatically in the few days before it.

As noted above, we identified queries for recipes and compared these recipes from 14 days before the first CRQ with recipes from 14 days after it. Table 2 shows the recipes and ingredients that increased in searches and those that decreased.

**Table 2:** Recipes and food ingredients that increased and those that decreased in the 14 days after the first celiac-related query, compared with the preceding 14 days.

Category	Increased	Decreased
Foods	Gluten-free pie crust	Honey cake
	Gluten-free pumpkin bread	Earthquake cake
	Gluten-free banana bread	Cucumber salad
	Gluten-free peanut butter cookies	Egg salad
	Gluten-free chocolate chip cookies	Pasta salad
	Gluten-free pancakes	Broccoli salad
	Roasted pumpkin seeds	Fish tacos
	Pumpkin soup	Ratatouille
	Cinnamon rolls	Tomato pie
	Pumpkin muffins	Tuna noodle casserole
Ingredients	Bean flour	All-purpose flour
	Brown rice flour	Dark rum
	White rice flour	Anisette
	Potato starch flour	Gin
	Rice flour	Serrano chile
	Xanthan gum	Peach schnapps
	Soy flour	Cherry
	Shortening	Pickles
	White sugar	Gelatin
	Ground walnuts	Sunflower seeds

## Discussion

In this analysis of search engine queries, we found that symptoms known to be associated with celiac disease were searched for in the days preceding a first-time search for celiac disease or gluten. The symptom most likely to be queried more before than after a celiac disease query was diarrhea, a common clinical manifestation of celiac disease [21]. Though a variety of other symptoms and associated conditions were also queried before a CRQ, there was no combination of terms that resulted in an area under the curve of high discriminatory value. This lack of a discriminatory symptom set is in contrast to a prior analysis of this search engine investigating symptoms preceding a diagnosis of pancreatic cancer, which found that a set of search terms can identify 5% to 15% of patients with likely pancreatic adenocarcinoma while maintaining a low false-positive rate [13]. The lack of a consistent set of symptoms preceding interest in celiac disease or gluten-related disorders is congruent with a recent study evaluating medical records that found that clinical manifestations and associated diseases were largely ineffective at distinguishing patients with and without celiac disease [22]. Case finding (as opposed to population screening) is a widely accepted approach to identifying patients with celiac disease; however, a symptom-based approach appears to be unable to effectively distinguish patients with celiac disease from the general population, and this is borne out by our analysis.

The majority of patients with celiac disease now present without diarrhea and instead with other intestinal or extraintestinal symptoms [8]. Nevertheless, no single nonclassical manifestation is more common than diarrhea as a presenting feature, and thus, the plurality of patients have diarrhea, which might account for this symptom being the most likely to be mentioned before a CRQ [8]. Among the so-called nonclassical presentations, we found that queries for bloating and gastroesophageal reflux were associated with subsequent sustained queries for celiac disease. Among extraintestinal symptoms, headache, anxiety, depression, and attention-deficit hyperactivity disorder were associated with subsequent CRQs, raising the possibility that neuropsychiatric symptoms are a more prominent set of clinical features in celiac disease than is generally recognized. Patients with celiac disease have a greater risk of health care visits for headache both before and after celiac disease diagnosis [23]. Most studies have also found an association between both anxiety and depression and celiac disease [24], and the presence of depression appears to modify the relationship between adherence to the gluten-free diet and the severity of celiac disease–related symptoms [25]. Our findings suggest that these neuropsychiatric symptoms may be a prominent feature among individuals before they seek celiac disease testing.

In addition to the known intestinal and extraintestinal conditions and associated diseases, our analysis also yielded unexpected associations with subsequent CRQs, including cough, asthma, bleeding, influenza, itch, colitis, and Alzheimer disease (Table 1). Though cough and asthma are not thought to be a common manifestation of celiac disease, patients with celiac disease are somewhat more likely to have asthma [26], and several conditions that feature cough are also associated with celiac disease, including pneumococcal pneumonia and influenza [27,28]. Itch may be associated with celiac-specific queries because of dermatitis herpetiformis, a gluten-induced blistering rash that can be intensely pruritic [29]. Colitis may be associated with celiac-associated queries because of the known association between celiac disease and lymphocytic and collagenous colitis, 2 forms of microscopic colitis that may improve after the adoption of a gluten-free diet [30].

To our knowledge, this is the first study to analyze individual search engine data to identify antecedent symptoms in those who subsequently express an interest in celiac disease. A prior study analyzing regional patterns of Google searches for the gluten-free diet found that location-derived sociodemographic factors such as median income and proportion of residents who are non-Hispanic white were associated with an increased rate of searches for the gluten-free diet as compared with other diets [31]. In this study, we were able to analyze individual-level search data, allowing us to draw inferences about the variety of symptoms that precede awareness of the celiac disease or gluten as a possible underlying cause. The use of search engine queries allows us to evaluate symptoms that may be embarrassing for individuals to report to health care practitioners or on a traditional questionnaire [12]. Another strength of this study was its large sample size, encompassing over 6500 individuals who exhibited a sustained interest by performing a CRQ over more than 1 day.

This study also has a number of limitations. We are unable to distinguish diagnosed celiac disease from those individuals merely suspecting celiac disease. We attempted to mitigate against this by analyzing transient versus persistent interest, as those with persistent interest are more likely to have received a diagnosis of celiac disease; nevertheless, search engine analysis is unable to distinguish celiac disease from nonceliac gluten sensitivity. Though self-identified queries are rare, they can be useful to validate more widely used queries, and we did find that CRQs were highly correlated with a self-identified celiac query (eg, “I have celiac”) when compared with the general population; nevertheless, avoidance of gluten is far more common than diagnosed celiac disease [3]. Individuals with nonceliac gluten sensitivity have beliefs and attitudes that differ from those with celiac disease with regard to the health effects of gluten, the safety of genetically modified organisms, and other issues [32]. Our findings of multiple differences in queried recipes, including a rise in gluten-free baked goods, suggest that regardless of their celiac disease status, users with a CRQ are changing their dietary habits, at least in the short term. Regulations prohibit analysis of search engine query data beyond 18 months, which is considerably shorter than the latency period reported between symptom onset and celiac disease diagnosis (a mean of 11 years) in questionnaire studies [9].

In conclusion, in this analysis of celiac-related internet queries, we found an increase in antecedent searches for symptoms known to be associated with celiac disease such as diarrhea, bloating, and weight loss; a rise in searches for depression and anxiety; and an increase in symptoms that are associated with celiac disease but may not be reported to health care providers. We also found that the protean clinical manifestations of celiac disease are reflected in the diffuse nature of antecedent internet queries, underscoring the challenge of effective case-finding strategies. Future studies should investigate the unexpected associations found with CRQs in this study as well as the prevalence and natural history of neuropsychiatric symptoms in patients at the time of celiac disease diagnosis.

## Abbreviations

CRQ: celiac-related queries
QR: query ratio
